# Laudatio zum 100. Geburtstag von Prof. Dr. Klaus Steigleder

**DOI:** 10.1111/ddg.15696_g

**Published:** 2025-03-07

**Authors:** Hans F. Merk, Thomas Krieg, David R. Bickers

Was waren die wichtigsten Ereignisse in der Dermatologie vor 100 Jahren? In Deutschland beschrieb in diesem Jahr der Internist und Dermatologe Viktor Klingmüller eine besondere Form der Akrozyanose, die er als Erythrocyanosis crurum puellarum bezeichnete; 1925 wurde der erste Dermatologische Lehrstuhl an der Hautklinik in Münster gegründet; Hermann Fabry junior, der später die Leitung der Hautklinik in Bochum übernahm, wurde 1925 geboren. Dieses Jahr war auch das Geburtsjahr von Hellmut Ippen, der als Oberarzt in Düsseldorf die Phlebotomie als erstes wirksames Behandlungsprinzip bei Porphyria cutanea tarda einführte und später Direktor der Hautklinik Göttingen wurde sowie von Hagen Tronnier, der 1972 die Hautklinik in Dortmund übernahm. Es scheint also, dass dieses Jahr für die Dermatologie, insbesondere für die Kliniken in Deutschland und in Nordrhein‐Westfalen von besonderer Bedeutung gewesen ist.

Gekrönt wurde dieses dann durch die Geburt von Gerd Klaus Steigleder, der als Dermatologe eine hohe internationale Bedeutung erlangte, der das Feld der dermatologischen Histopathologie für lange Zeit prägte und der am 25. Januar 2025 nun mit seiner Familie seinen 100 Geburtstag feiert.

Viele Studierende der Medizin haben Gerd Klaus Steigleder als Autor der gut geschriebenen kompakten blauen Thieme Bücher zur Dermatologie kennen gelernt. Unter den Dermatologen war er als Herausgeber der „Zeitschrift für Hauterkrankungen“ – dem Vorläufer der „JDDG“, als langjähriger Direktor der Kölner Hautklinik und Lehrstuhlinhaber, Generalsekretär und Präsidenten der Deutschen Dermatologischen Gesellschaft fast jedem ein Begriff. Er, Schüler von Oscar Gans und in der Tradition von Paul Gerson Unna, war ein herausragender klinischer Dermatologe, der frühzeitig den Weg in die USA gesucht hat. Dort begeisterte er sich für die moderne Dermatologie, die er weiterentwickelt hat und die ihn zu einem der großen, namhaften Dermatologen des 20. Jahrhunderts machte. Er wurde mit vielen nationalen und internationalen Preisen, einschließlich der Herxheimer‐Medaille, ausgezeichnet.

Mit 17 Jahren legte er das Abitur ab, entschied sich, Medizin zu studieren und schlug dann einen Weg nach dem Studium ein, der für seinen späteren Lebensweg prägend war. So konzentrierte er sich besonders auf die so wichtige Morphologie, die Anatomie und Histologie, aber auch auf die physiologische Chemie und die Innere Medizin. Schließlich ging er ab 1950 in die Dermatologie, wurde Facharzt und sehr schnell mit 27 Jahren der jüngste Priv.‐Doz. der Medizin. Die dermatologische Ausbildung erfuhr er bei Oscar Gans, dem damaligen Direktor der Frankfurter Hautklinik. Oscar Gans war Schüler von Paul Gerson Unna und durch sein Standardwerk zur „Histologie der Hauterkrankungen“ ein besonders renommierter Dermatohistologe. Gerd Klaus Steigleder interessierte sich nicht zuletzt vor dem Hintergrund seiner früheren Tätigkeit in der Anatomie für dieses Gebiet, bearbeitete zusammen mit Oscar Gans die zweite Auflage dieses Buches und habilitierte sich 1952 über „Die Struktur und Funktion der Akanthose“. Langwierige, endlos‐erscheinende Entscheidungsprozesse liebte GK‐Steigleder überhaupt nicht und so nimmt es nicht Wunder, dass er sich kurze Zeit nach der Habilitation zu einem Studienaufenthalt in den USA entschied. Dort hatte er die Gelegenheit, sich von 1956 bis 1957 der Arbeitsgruppe von Stephen Rothman in der Hautklinik in Chicago anzuschließen. Stephen Rothman repräsentierte damals bereits eine Forschungsrichtung, die sich nicht ausschließlich auf die Morphologie konzentrierte, sondern funktionell orientiert war. Das Zusammenbringen dieser unterschiedlichen Expertisen, der Histologie und einer experimentellen funktionellen Dermatologie mit dem Ziel, Morphologie und Funktion gemeinsam zu verstehen und zu verbinden, war damals neu und führte zu vielen überraschenden Erkenntnissen in der Forschung, die dann schnell Eingang in die Lehrbücher fanden. Diese Arbeiten waren so erfolgreich, dass Gerd Klaus Steigleder viele Jahre in den USA blieb und 1959 in der von Carl Truman Nelson geführten Hautklinik der Columbia Universität New York die Leitung der Dermatohistologe, einschließlich Histologie der Muskelerkrankungen, übernahm.

Hier wurden von ihm wesentliche neue Konzepte aufgrund morphologischer und funktioneller Studien einschließlich des Konzepts vom „Enzymmantel der Haut“ gelegt und es folgten Erstbeschreibungen einschließlich des Menkes‐Steigleder‐Syndrom. Schließlich zeichnete sich bald auch die Möglichkeit ab, dass Gerd Klaus Steigleder sein weiteres Berufsleben in den USA verbringen würde. Allerdings wurde dann der ebenfalls in New York in der Klinik von Marion Sulzberger arbeitenden Franz Hermann auf den Lehrstuhl in Frankfurt berufen. Dieses veranlasste Gerd Klaus Steigleder mit ihm nach Deutschland zurückzukehren. Bald bot sich ihm die Möglichkeit, den dermatologischen Lehrstuhl in Heidelberg oder Köln zu übernehmen. Er entschied sich für die Universität zu Köln und wurde bereits mit 39 Jahren Direktor der Hautklinik in Köln. Damals begann dann eine besonders enge Beziehung der Dermatologie in Köln mit der Columbia Universität, die sich bis heute fortsetzt und zu immer wieder erneuerten Partnerschaften auf unterschiedlichen Ebenen führte.

In Köln leitete er als Direktor die Hautklinik sehr erfolgreich, prägte diese mit seinem Forschungsfeld und wurde einer der einflussreichsten deutschen Dermatologen. GK‐Steigleder war ein hervorragender Mentor und baute seine eigene dermatologische Schule auf (Abbildung 1). Mehrere Mitarbeiter habilitierten sich in Köln und wurden später auf Lehrstühle berufen beziehungsweise wurden Klinikdirektoren wie C. Orfanos (Berlin), WP. Hermann (Bremen), H. Pullmann (Lüdenscheid), W. Sterry (Berlin), H. Merk (Aachen) oder später dann auch M. Hertl in Marburg. Noch in den letzten Jahren der Klinikleitung warb GK‐Steigleder selbst zusammen mit Physikern in Bochum ein DFG‐Projekt zur Bestimmung der Verteilung von Metallen in Psoriasishaut mittels PIXE‐Analyse ein. GK‐Steigleder wurde frühzeitig in die nationale Akademie der Wissenschaften gewählt und nahm auch nach seiner Emeritierung an den Sitzungen der dermatologischen Sektion der Leopoldina regelmäßig teil. Auch nach seiner Emeritierung blieb er der Dermatologie treu und führte eine Praxis. Lange Zeit nahm er an allen Fortbildungsveranstaltungen in Köln teil und stellte immer die schwierigsten Fragen,

Am 25. Januar 2025 vollendet Prof. Steigleder nun sein 100. Lebensjahr. Dazu gratulieren wir Ihnen herzlich verbunden mit dem Wunsch, dass Sie der dermatologischen Familie sowie Ihrer Frau Inge Steigleder, den Kindern, Enkelkindern und inzwischen auch Urenkeln noch viele Jahre mit Rat und Tat zur Verfügung stehen.

## DANKSAGUNG

Open access Veröffentlichung ermöglicht und organisiert durch Projekt DEAL.

**ABBILDUNG 1:**
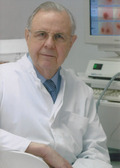
Prof. Dr. Gerd Klaus Steigleder.

